# Structural basis for the target specificity of actin histidine methyltransferase SETD3

**DOI:** 10.1038/s41467-019-11554-6

**Published:** 2019-08-06

**Authors:** Shaobo Dai, John R. Horton, Clayton B. Woodcock, Alex W. Wilkinson, Xing Zhang, Or Gozani, Xiaodong Cheng

**Affiliations:** 1 Department of Epigenetics and Molecular Carcinogenesis, The University of Texas MD Anderson Cancer Center, Houston, TX 77030 USA; 2 Department of Biology, Stanford University, Stanford, CA 94305 USA

**Keywords:** Transferases, X-ray crystallography, Methylation, Enzyme mechanisms

## Abstract

SETD3 is an actin histidine-N_3_ methyltransferase, whereas other characterized SET-domain enzymes are protein lysine methyltransferases. We report that in a pre-reactive complex SETD3 binds the N_3_-protonated form (N_3_-H) of actin His73, and in a post-reactive product complex, SETD3 generates the methylated histidine in an N_1_-protonated (N_1_-H) and N_3_-methylated form. During the reaction, the imidazole ring of His73 rotates ~105°, which shifts the proton from N_3_ to N_1_, thus ensuring that the target atom N_3_ is deprotonated prior to the methyl transfer. Under the conditions optimized for lysine deprotonation, SETD3 has weak lysine methylation activity on an actin peptide in which the target His73 is substituted by a lysine. The structure of SETD3 with Lys73-containing peptide reveals a bent conformation of Lys73, with its side chain aliphatic carbons tracing along the edge of imidazole ring and the terminal ε-amino group occupying a position nearly identical to the N_3_ atom of unmethylated histidine.

## Abstract (web summary)

SETD3 is the first known metazoan protein histidine methyltransferase but the molecular basis for its target specificity is unclear. Here, the authors elucidate the structural and molecular determinants for the histidine specificity of SETD3.

## Introduction

Protein lysine methylation, particularly histone lysine methylation, has been extensively studied for the past two decades, ever since the discovery of SUV39H1 as the histone H3 lysine 9 specific methyltransferase (MTase)^1^ (reviewed in refs. ^2–9^). It was the sequence similarity to a plant Rubisco large subunit MTase (LSMT)^10,11^, which led to the discovery that the SET domain of SUV39H1 possesses methylation activity^1^. In humans, about half of the 55 SET-domain containing proteins methylate lysine residues on histone and/or non-histone protein substrates^12^. The literature on SET-domain-catalyzed protein lysine methylation has demonstrated that these MTases frequently have high substrate specificity due to recognition of the various sequences surrounding the target lysine and distinct product specificity for the number of methyl groups (one, two, or three) being transferred to the target lysine^13–20^. Recently, SETD3 was discovered as the first metazoan protein (actin) histidine MTase^21–23^, representing a SET domain family member that methylates a target residue other than lysine, thus broadening the possible target residues that can be methylated by SET domain proteins. This raises the previously unappreciated question of target specificity, i.e., how do SET-domain MTases discriminate between histidine and lysine in the active-site pocket?

Among the structurally characterized SET domain proteins, SETD3, human SETD6^24,25^ and Rubisco LSMT^26^ share global structural similarity (Supplementary Fig. 1a–c). In addition to the N-terminal catalytic SET domain, the three protein MTases share a C-terminal elongated helical domain, which in the case of LSMT interacts with the part of Rubisco away from its target lysine^27^. Despite the overall structural similarity, the sequence identity among the three proteins is <10% (Supplementary Fig. 1d), and is scattered through the entire region, including the invariant residues involved in structural integrity, intra-molecular interactions in the interior of the molecule conferring stability and those with conserved function of binding the methyl donor *S*-adenosyl-l-methionine (SAM) (Supplementary Fig. 1e). On the substrate-binding surface, the residues are diverse (Supplementary Fig. 1f), reflecting the fact that the three enzymes act on three very distinct substrates—the large subunit of Rubisco by LSMT, RelA subunit of nuclear factor NFκB by SETD6, and actin by SETD3—with limited sequence similarity immediately surrounding the methylation target (Supplementary Fig. 1g).

Here we elucidate the structural and molecular determinant(s) of target specificity of histidine vs. lysine in the active site of SETD3. To facilitate the methyl transfer reaction, SETD3 provides a local environment that promotes deprotonation of the target nitrogen N_3_ of histidine (and concomitant protonation of N_1_). While the active-site residues maintain their conformations in the pre-reactive and post-reactive complexes, Asn255 forms the crucial hydrogen bond to the protonated N_1_ nitrogen of histidine. The asparagine-to-valine (N255V) or alanine (N255A) mutants have reduced histidine methylation activity. More interestingly, N255A and N255V mutants have increased lysine methylation activity compared to wild type SETD3. These results suggest a possible evolutionary trajectory between lysine-specific and histidine-specific SET domain MTases.

## Results

### SETD3 methylation of histidine

**Fig. 1:**
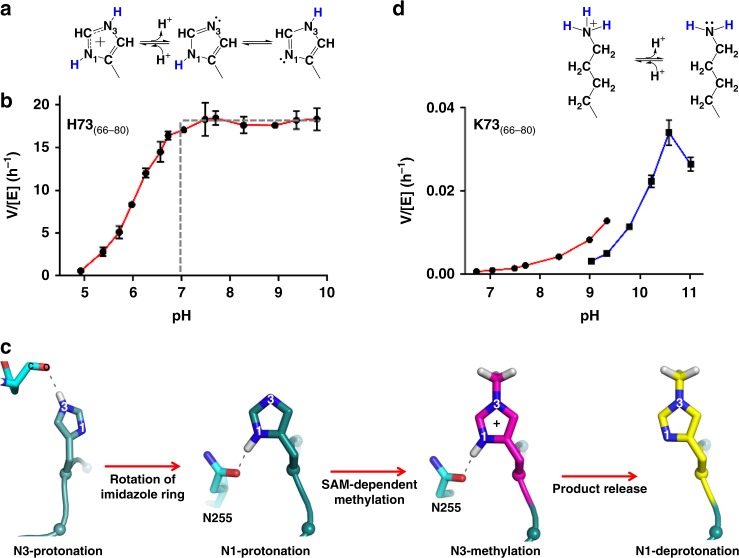
SETD3 is active on methylation of histidine and lysine residues with different optimal pH. **a** The state of protonation of N_1_ and N_3_ nitrogen atoms of the imidazole ring of histidine. **b** SETD3 activity on H73_(66–80)_ peptide as a function of pH. The buffers contain a mixture of 10 mM citric acid and 10 mM BisTris propane (CBTP). **c** A model of histidine-N_3_ methylation in the active-site of SETD3 involves N_3_-protonation, rotation of imidazole ring, N_1_-protonation, N_3_-methylation, product release, and N_1_-deprotonation (for example, in or near physiological pH). **d** Chemical structure of deprotonated lysine near pH 10 (top panel), and SETD3 activity on K73_(66–80)_ as a function of pH. The buffers used were CBTP for pH below 9 and 20 mM glycine for pH above 9. Data represent the mean ± SD of two independent determinations (*N* = 2) performed in duplicate. Source data are provided as a Source Data file

The imidazole ring of histidine residue contains two nitrogen atoms (N_1_ near the backbone and N_3_ farther from the backbone) and both of them can be protonated (Fig. 1a). Most (if not all) SAM-dependent MTases use the classic S_N_2 reaction mechanism^28^, which requires the target nitrogen be in a deprotonated state. SETD3 is in agreement with this mechanism as it has an optimum pH of 7 and above (Fig. 1b), when the imidazole ring (with a typical p*K*_a_ value near 6) is uncharged. However, the remaining proton is in equilibrium between the tautomers and can reside on either nitrogen (N_1_-H or N_3_-H) (Fig. 1a). To facilitate the methyl transfer reaction, the enzyme needs to provide a local chemical environment that stabilizes N_1_ nitrogen in the protonated form so that the target nitrogen N_3_ is in the deprotonated form. The X-ray diffraction technique does not provide direct information on hydrogen atoms^29^. However, our structures are determined at resolutions between 1.75 and 2.09 Å (Supplementary Table 1) and the known chemical natures of interacting groups allow us to predict the presence of a proton between two interacting atoms forming a hydrogen bond (H-bond).

Previously, we solved a ternary complex structure including SETD3, the actin peptide residues 66–80 with the target histidine 73 (H73) in the middle, and *S*-adenosyl-l-homocysteine (SAH)—the reaction product of the methyl donor SAM^22^. However, this peptide substrate–cofactor product complex structure does not represent a particular state along the methylation reaction pathway, so we characterized two complex structures representing states before and after the methylation reaction.

**Fig. 2:**
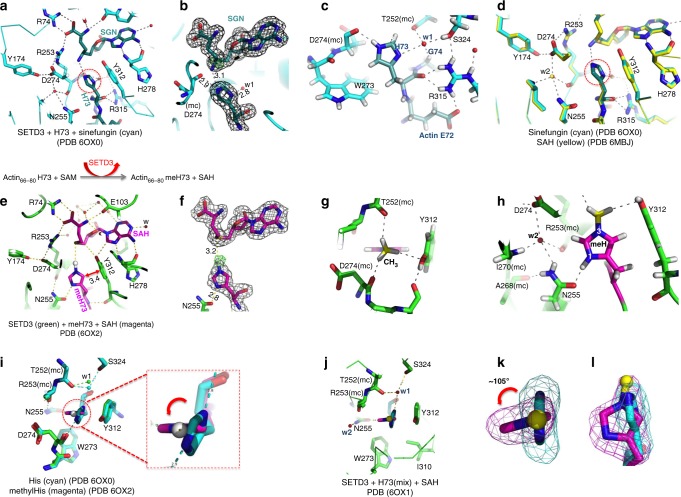
Conformational rotation of actin H73 in pre-reactive and post-reactive complexes. **a** A pre-reactive complex of SETD3, H73 peptide, and sinefungin (SGN) (PDB 6OX0). **b** Omit electron density maps, respectively, for sinefungin and H73, contoured at 5.0*σ* above the mean. **c** A model with hydrogen atoms (light gray) included for H73 and its immediate surrounding (nitrogen, blue; oxygen, red; carbon, cyan). **d** Superimposition of Sinefungin and SAH-mediated complex structures. **e** A post-reactive complex of SETD3 formed after going through the methylation reaction (PDB 6OX2). **f** Omit electron density maps, respectively, for SAH, methyl H73 (gray) and the methyl group (green), contoured at 5.0*σ* above the mean. **g** The methyl group (CH_3_) of methyl H73 forms three C–H•••O type of H-bonds. **h** Asn255 forms a H-bond with the N_1_ nitrogen of H73. **i** Superimposition of H73 and methyl H73 from pre-reactive and post-reactive complexes. **j** A mixture of H73 and methylH73 in the complex structure (PDB 6OX1). **k**, **l** Two orthogonal views showing omit electron densities, respectively, for H73 (cyan) and methylH73 (magenta), contoured at 5.0*σ* above the mean

First, we used the SAM analog sinefungin (adenosyl ornithine) to prepare a complex mimicking the pre-reactive state (Fig. 2a). Like SAM, sinefungin also carries a formal positive charge, but does not support the methyl transfer, and thus functions as an inhibitor (Supplementary Fig. 2). The target histidine’s imidazole ring is nearly parallel to the aromatic ring of Tyr312, and, to maximize the H-bond potential of both nitrogen atoms, the imidazole ring is positioned such that N_3_ donates a proton to the main-chain carbonyl oxygen atom of Asp274 (N_3_–H•••O = C), while the N_1_ accepts a proton from a water molecule (numbered as w1) (Fig. 2b). This water molecule is coordinated adjacent to the histidine-binding pocket, and is saturated with four H-bonds (Fig. 2c), three of which are at a traditional H-bond distance from the interacting atoms: the N_1_ atom of the histidine (2.8 Å), the main-chain carbonyl oxygen atom of Thr252 (2.6 Å) and the side-chain hydroxyl oxygen atom of Ser324 (2.7 Å). The fourth H-bond to w1 is weak and is within a distance of 3.4–3.5 Å from the main-chain carbonyl oxygen of the target histidine, the Cα atom of actin residue G74, or one of the guanidino nitrogen atoms of Arg315 (Fig. 2c). The conformation of unmethylated histidine in the sinefungin complex is similar to that in the previous determined structure of the SETD3–SAH–H73 complex (Fig. 2d).

Second, we incubated SETD3 with actin peptide in the presence of a molar excess of SAM (at pH 8.0) and crystallized the post-reactive product complex, i.e., the methylated histidine and SAH (Fig. 2e). The methyl group at the N_3_ position of methylhistidine is 3.2 Å away from the sulfur atom of SAH (Fig. 2f), where the transferable methyl group was attached before the reaction, and makes three weak C–H•••O hydrogen bonds^26^, respectively, with the main chain carbonyl oxygen atoms of Thr252 and Asp274 as well as the hydroxyl oxygen of Tyr312 (Fig. 2g). The methylated imidazole ring is perpendicular to the aromatic ring of Tyr312, and the nitrogen atom N_1_ is within H-bond distance from the side chain of Asn255 (Fig. 2h). The side chain amide group of asparagine is capable of both donating an H-bond (via the amino nitrogen −NH_2_), and accepting an H-bond (via the carbonyl oxygen −C=O). The current configuration of Asn255 appears to serve as a proton acceptor from the protonated N_1_ (N_1_–H•••O=C) of methylated H73; and to donate two protons to the main chain carbonyl oxygen atom of Ala268, and to a water molecule (numbered w2). The water (w2), in turn, forms three additional H-bonds with the main chain amide nitrogen atom of Ile270, one of the side chain carboxylate oxygen atoms of Asp274, and the main chain carbonyl oxygen atom of Arg253 (Fig. 2h). This suggests that immediately after methyl transfer, the methylated histidine is positively charged with a protonated N_1_ (N_1_-H) and a methylated N_3_ (N_3_-CH_3_) and forms a cation–π interaction with Tyr312. After being released from the active site, the methylhistidine could become neutral depending on the local pH environment (Fig. 1c). The main difference between the pre-reactive and post-reactive complex structures is that the imidazole ring of methylated His73 rotated away from that of histidine (Fig. 2i).

Third, to verify that the target histidine, before and after the methyl transfer reaction, can adopt two different conformations in the active site, we reduced the concentration of SAM in the reaction, which resulted in a mixture of methylated and unmethylated peptides, and followed with crystallization. Indeed, the active-site density for the histidine residue can be modeled by two ring conformations with a rotation of ~105° (Fig. 2j–l), whereas the conformations of residues lining the active-site pocket remain unchanged. The position of the target N_3_ atom and its relative distance to the cofactor in the two conformations are virtually identical. We think the primary reason for the rotation is to switch the proton from N_3_-H to N_1_-H, ensuring the target atom N_3_ is deprotonated prior to the methyl transfer (Fig. 1c). As a result, the deprotonated N_3_ nitrogen carrying a lone pair of electrons attacks, in a nucleophilic manner, the positively charged methylsulfonium of SAM.

**Fig. 3:**
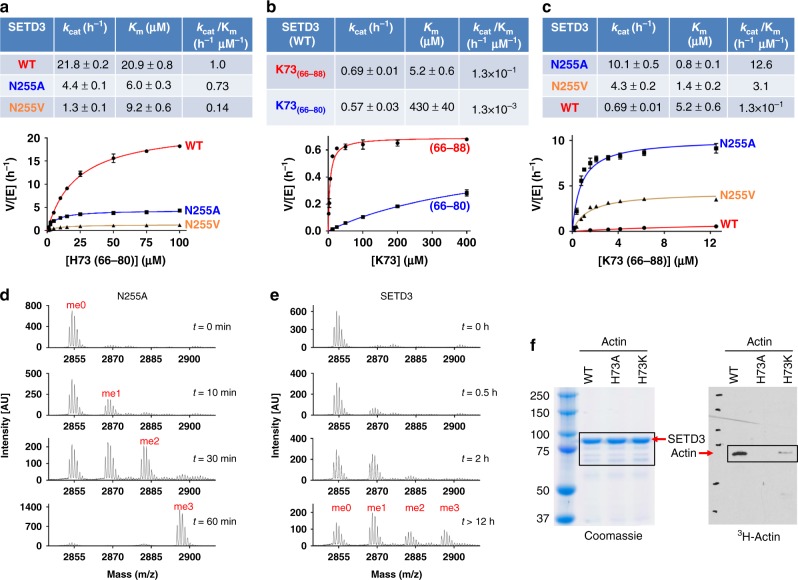
Kinetics of SETD3 on H73 and K73 peptides. **a** Comparison of activities of three variants (WT, N255A, and N255V) on H73_(66–80)_ peptide. The assays were performed at room temperature (~22 °C) and pH 8.0 using [WT] = 0.18 μM, [N255A] = 0.18 μM, and [N255V] = 0.72 μM. **b** Comparison of SETD3 WT activities on two K73 peptides (residues 66–80 and 66–88). The assays were performed at 37 °C and pH 10.5 using [WT] = 3 and 15 μM, respectively, for the long and short peptides. **c** Comparison of activities of three variants (N255A, N255V, and WT) on K73_(66–88)_ peptide. The assays were performed at 37 °C and pH 10.5 using [N255A] = 90 nM, [N255V] = 0.72 μM and [WT] = 4 μM. Data represent the mean ± SD of *N* number of independent determinations (*N* = 3 for the WT and *N* = 2 for the mutants) performed in duplicate. Source data are provided as a Source Data file**. d**, **e** Mass spectrometry analysis of K73_(66–88)_ methylation kinetic showing representative spectra at various time points for N255A variant (panel **d**) and SETD3 WT enzyme (panel **e**). The assays were performed at 37 °C and pH 10.5 using 3 μM for both N255A and WT. **f** In vitro methylation reactions with SETD3, indicated full-length actin proteins, and radiolabeled methyl donor (^3^H-SAM). Right, Reactions were resolved by SDS–PAGE and activity was visualized by autoradiography. Left, Coomassie blue staining was used as loading control

The active-site pocket that houses the target histidine is lined with the side chains of Asn255 (Phe in SETD6 and LSMT), Trp273 (Ala in SETD6 and LSMT), Ile310 (Asn in SETD6 and Ile in LSMT), Tyr312, and main-chain atoms of Thr252 and Arg253 (Fig. 2i, j). Among them, only Tyr312 is conserved in SETD6 and LSMT, and the hydroxyl group of Tyr312 is hydrogen bonded to the backbone carbonyl oxygen of Cys276 and is snug between the two moieties of the cofactor (SAM/SAH/sinefungin) (Fig. 2a, e). The Y312A mutant in SETD3 has residual activity^22^, but the corresponding Y285A mutation in SETD6 abolished its enzymatic activity^25^, and the corresponding Y283F mutation in DIM-5 (a Neurospora histone H3 lysine 9 MTase) lost its SAM binding and thus activity^30^. We hypothesized that the non-conserved Asn255 and its H-bond to the N_1_ atom of the imidazole ring is important for orienting and shifting the proton from N_3_-H to N_1_-H (Figs. 1c and 2h). Substituting Asn255 with alanine (N255A) or valine (N255V) resulted in reduced *k*_cat_ values of 4.4 h^−1^ (N255A) or 1.3 h^−1^ (N255V) from 22 h^−1^ of wild-type (WT) SETD3 (Fig. 3a), supporting the notion that *k*_cat_ could be substantially slower if the substrate was a mix of the two protonation states.

### SETD3 methylation of lysine

Next, we asked whether SETD3 is capable of methylating a lysine (H73K) in the context of the same actin peptide residues 66–80. Under the conditions that were optimized for the H73 peptide (pH 8.0), we observed no activity on the K73-containing peptide (Supplementary Fig. 3a). We reasoned that a different condition might exist for a lysine substrate, mainly because of its typical p*K*_a_ value of ~10, and as that other biochemically characterized SET domain lysine MTases (DIM-5, LSMT, and SETD6) showed maximal in vitro activity at approximately pH 10^24,30,31^. After adjusting the pH (Fig. 1d), temperature (Supplementary Fig. 3b-c), and concentrations of enzyme, substrate, and SAM, SETD3 is active on K73-containing peptide (residues 66–80) with a *k*_cat_ = 0.6 h^−1^ and *K*_m_ = 430 μM under the laboratory condition of pH 10.5 at 37 °C (Fig. 3b). Nevertheless, SETD3 is a slow and low-affinity enzyme with the K73_(66–80)_ substrate, exhibiting a maximum reaction rate ~40-fold slower (*k*_cat_ = 0.6 h^−1^ on K73 vs. 22 h^−1^ on H73), and an affinity for the substrate ~20-fold weaker, than for the H73 substrate (*K*_m_ = 430 vs. 21 μM). In other words, SETD3 shows more than 1000-fold loss in catalytic efficiency (comparing *k*_cat_/*K*_m_ value of ~1 h^−1^ μM^−1^ for H73_(66–80)_ and 1.3 × 10^−3^ h^−1^ μM^−1^ for K73_(66–80)_ peptides) driven by the change of the target residue from H73 to K73 (Fig. 3a, b). It is thus no surprise that we did not observe the K73-containing peptide (residues 66–80) after attempting to grow co-crystals.

**Fig. 4:**
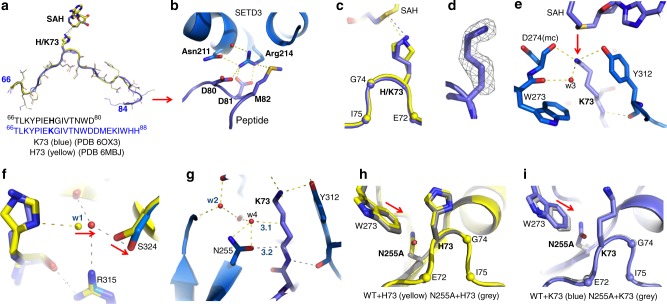
Structure of SETD3 in complex with K73 in the active site. **a** Superimposition of H73 (yellow; PDB 6MBJ) and K73 (blue; PDB 6OX3) containing peptides in respective complex structures. **b** Arg214 of SETD3 interacts with D80, D81, and M82 of peptide substrate. **c** Superimposition of His73 (yellow; PDB 6MBJ) and Lys73 (blue; PDB 6OX3) in the active site of SETD3. **d** Omit electron density map for the side chain of K73 contoured at 5.0*σ* above the mean. **e** Deprotonation of K73 of peptide and Tyr312 of SETD3 (a conserved residue in SET domain proteins) would facilitate lysine methylation. **f** The water molecule w1 moves away from K73. **g** Asn255 interacts with the main-chain atom of K73. **h** Superimposition of WT SETD3 (yellow; PDB 6MBJ) and N255A variant (gray; PDB 6OX4) in complex with H73 peptide. **i** Superimposition of WT SETD3 (blue; PDB 6OX3) and N255A variant (gray; PDB 6OX5) in complex with K73 peptide

In order to further grasp the measurable activity of SETD3 on K73-containing peptides, we attempted to improve the enzyme affinity (*K*_m_ value) by increasing the peptide length to actin residues 66–88 (as used by Guo et al.^23^). Indeed, the lengthened peptide decreased the *K*_m_ value (increased affinity) to 5.2 μM (pH 10.5) against K73_(66–88)_ peptide, which is about four-fold better than that of the H73_(66–80)_ peptide (Fig. 3b). The improved *K*_m_ value resulted in an increased catalytic efficiency on K73 by a factor of ~100 (*k*_cat_/*K*_m_ = 1.3 × 10^−3^ and 1.3 × 10^−1^ h^−1^ μM^−1^ for the short and long peptides, respectively) (Fig. 3b). The longer K73-containing peptide readily crystallized with SETD3 and SAH (Fig. 4a).

The structure of SETD3 in complex with K73_(66–88)_ peptide and SAH is highly similar to those of the SAM-mediated, SAH-mediated, or sinefungin-mediated H73 complexes, with pairwise comparison of ~0.2 Å across 866 pairs of Cα atoms (two complexes per crystallographic asymmetric unit in *P*2_1_ space group). The conformation of K73-containing peptide superimposes well with that of H73-containing peptide, except for the newly added C-terminal peptide residues (particularly D81 and M82; single letter-code for peptide and three-letter code for SETD3), which provide additional inter-molecular interactions (Fig. 4a). Peptide residues D80 and D81 form charged interactions with Arg214 of SETD3 (which also forms an intra-molecular polar interaction with Asn211), while peptide residue M82 packs against the guanidine group of Arg214 (Fig. 4b). Thus, a positively charged Arg214 of SETD3 is buried in the enzyme–peptide interface and is critical for stabilizing a network of inter-molecular and intra-molecular interactions that likely confer enhanced affinity. The enhanced enzyme–peptide interaction might force the lysine side chain of K73 into the active site pocket, which is not deep enough for a linear aliphatic chain of lysine (see Fig. 2f of Wilkinson et al.^22^). Interestingly, the side chain of K73 adopts a bent conformation, such that its side chain aliphatic carbons trace along the edge of the superimposed imidazole ring, and the terminal ε-amino group occupies a position nearly identical to the N_3_ atom of unmethylated histidine (Fig. 4c, d). The positively charged ε-amino group is effectively stabilized by the partial negative dipole of main-chain carbonyl oxygen atom of Asp274, hydroxyl oxygen atom of Tyr312, and a water molecule (numbered w3), which is connected to the main-chain carbonyl oxygen atom of Trp273 (Fig. 4e). In addition, the ε-amino group is 3.4 Å away from the sulfur atom of SAH, where a transferable methyl group would be attached. Thus, at pH 10, the ε-amino group of the target lysine and the hydroxyl group of Tyr312 (which would all have typical p*K*_a_ values of ~10) should be partially deprotonated. The deprotonated Tyr312 (O^−^) and the water molecule w3 could thereby facilitate the deprotonation of the target lysine amino group and undergo a nucleophilic attack on the positively charged SAM methylsulfonium. We note that most of the structurally characterized protein lysine MTases have a linear aliphatic chain of lysine substrate, except a mixture of linear and bent conformations was observed in the active site of SETD6^24^, the closest relative of SETD3.

Comparing K73 to H73 in the active site, we observed two subtle, but important differences. (1) The aforementioned H-bond between histidine N_1_ atom and a water molecule (w1) in the pre-reactive complex is no longer needed in the case of K73, and the w1 water shifted away from the lysine and was accompanied by the side chain movement of Ser324 (Fig. 4f). (2) The H-bond interaction—between the N_1_ atom of histidine and Asn255 in the post-reactive complex—is also absent in the K73 complex, and a water molecule (numbered w4) occupied the space instead (Fig. 4g). Both the oxygen atom of the Asn255 amide group, and the water molecule w4, are ~3.2 Å away from the main-chain Cα atom and aliphatic side chain of K73. Eliminating these interactions might help release the tension of the bent lysine conformation. Indeed, both N255A and N255V have enhanced activity on K73 in the order N255A≫N255V>WT (N255) (Fig. 3c). This order of activity on K73 reflects the size (alanine vs. valine), as well as the potential of making H-bonds of residue 255 (asparagine). In contrast, the activity on H73 is in the reverse order (WT≫N255A>N255V) (Fig. 3a).

In addition, we determined the N255A mutant structures in complex with H73-containing and K73-containing peptides, respectively (Fig. 4h, i). Pairwise comparison between the WT and mutant enzyme complexed with the same peptide revealed identical overall structures with root-mean-square deviations of 0.4–0.5 Å across all Cα atoms. A small movement towards to the respective target residue (H73 or K73) is observed for Trp273 in the absence of side chain of Asn255 (Fig. 4h, i). With no H-bond potential with the imidazole ring, N255A mutant reduced activity on H73 methylation, whereas the extra space generated by removing the side chain of N255 might provide flexibility for multiple round of K73 methylation (see below).

Comparing to the WT SETD3, the variant N255A has significantly enhanced activity on K73 peptide (Fig. 3c). Thus, we asked how many methyl groups the variant enzyme is able to transfer to K73 (i.e., the product specificity of lysine methylation). We monitored the kinetics of product formation with the K73_(66–88)_ peptide as substrate, using MALDI-TOF mass spectrometry. Figure 3d, e shows representative spectra and time course for WT SETD3 and its variant N255A. We observed monomethyl lysine produced by the N255A variant at 10 min, dimethyl at 30 min, and a complete conversion of unmodified substrate to trimethyl lysine at 1 h (Fig. 3d). In contrast, WT SETD3 produced only monomethyl lysine at 2 h. After overnight incubation, substantial amounts of unmodified and monomethyl substrates remain, along with a small amount of dimethyl and tri-methyl products (Fig. 3e). In consistence with the peptide substrate, SETD3 exhibited activity on full-length recombinant actin-K73, albeit weaker than actin-H73 activity, in conditions more similar to those optimized for H73 methylation (pH 8.0, 30 °C) (Fig. 3f).

## Discussion

In conclusion, we investigated the determinant(s) of target specificity of histidine vs. lysine in the active site of SETD3. The enzyme has different optimized conditions for the in vitro methylation of histidine and lysine, mainly to prepare the substrates in a deprotonated state. The active site of SETD3 is constructed so that it provides a local chemical environment that enables the target histidine nitrogen N_3_ being deprotonated prior to methyl transfer. We also determined the structural basis of target specificity by engineering variants of SETD3 that have altered specificity. Taken together, SETD3 has a greater substrate specificity for recognition of long and ordered peptide, suggesting that SETD3 would inefficiently accommodate substrates divergent from actin. Once in the active site, Asn255 and its H-bond to the N_1_ atom of the imidazole ring make the histidine residue a preferred target. Eliminating the potential of forming H-bonds with an increased size of active site enables the N255A variant to be a competent trimethyl lysine MTase.

Previously, Guo et al. crystallized SETD3 in a ternary complex with a pre-methylated actin peptide and SAH (PDB 6ICT), where the methylated imidazole ring rotated by ~90° relative to that of the unmodified His73^23^. The observations between the two complexes with the methylated peptides (whether through chemical synthesis or enzyme driven reaction as described here) are generally in agreement, including the rotation of imidazole ring (though the degree of the rotation is not exactly the same) and the hydrogen bond between the N_1_ atom and Asn255. Only after comparing the two structures did we understand that imidazole ring rotation is necessary for appropriate target protonation in order to promote the methyl transfer reaction. The current study also suggests that the main reasons for low lysine methylation by SETD3 are lysine protonation and low binding affinity, rather than the size of binding pocket as suggested by Guo et al.^23^. Evidently, the pocket is compatible with lysine under the optimized conditions.

As we noted earlier, that the three SET domain proteins, plant LSMT and human SETD3 and SETD6, share a striking structural similarity throughout the entire protein (Supplementary Fig. 1). Given the restriction of Rubisco to plant species, the astonishing resemblance of these three protein MTases—acting on three different protein substrates and two very different target residues (lysine vs. histidine)—suggests that this particular fold of the protein MTase has been evolutionarily successful. However, four out of five residues that form active-site pocket are not conserved (Asn255, Trp273, Ile310, and Ser324 in Fig. 2j and Supplementary Fig. 1d). The fact that mutant variants N255A and N255V have altered their relative activities on histidine and lysine targets suggests that further adjustment/optimization in the active site could potentially generate variants that will selectively methylate lysine (or even other residues).

## Methods

### Purification of SETD3

Recombinant human SETD3 (pXC2003) was expressed as a GST-fusion^22^ in *Escherichia coli* BL21(DE3) CodonPlus^TM^ cells (Stratagene). The SETD3 mutants at residue 255 (N255A; pXC2093 and N255V; pXC2094) were generated with Q5 Site-Directed Mutagenesis Kit (New England Biolabs), confirmed by sequencing and the same protocol for expression and purification were followed. Briefly, the purification was conducted in a BIO-RAD NGC^TM^ system using a three-column chromatography of glutathione-sepharose, HiTrap Q-HP and Superdex 200. The GST tag was removed by PreScisson Protease (purified in-house). The purified SETD3 proteins at a concentration ~25–35 mg/ml were kept at −80 °C in storage buffer for future use [20 mM Tris (pH 8.0), 200 mM NaCl, 5% glycerol, and 0.5 mM tris (2-carboxyethyl) phosphine (TCEP)].

### Crystallography

An Art Robbins Phoenix Crystallization Robot was used to set up 0.4-μl sitting-drops at ~20 °C of the ternary complexes (~14 mg/ml or ~0.2 mM) plus the well solutions of 0.2 M ammonium acetate, 0.1 M sodium citrate tribasic dihydrate pH 5.6 and 30% (w/v) polyethylene glycol 4000. The complexes were prepared as following.

For the pre-reactive complex, SETD3 was mixed with sinefungin and H73 peptide (residues 66–80, Genscript) at a molar ratio of 1:4:5. The mutant N255A, in complex with SAH and H73 (residues 66–80) was generated similarly at a molar ratio of 1:4:5 and incubated in storage buffer on ice for 1 h.

For the post-reactive complex, SETD3 was first mixed with SAM and unmodified H73 peptide at a molar ratio of 1:10:5 and incubated at 4 °C in storage buffer overnight for the methylation reaction to happen, which generated SAH and the fully methylated H73 peptide. For partial methylation complex, SETD3, SAM, and the peptide was mixed at a ratio of 1:4:5 and incubated at 4 °C in storage buffer overnight.

For the SETD3 complex with SAH and actin H73K peptide (residues 66–88, Genscript), a molar ratio of 1:5:8 was used and incubated in storage buffer on ice for 8 h. The complex of the mutant N255A with SAH and K73 (residues 66–88) was formed at a molar ratio of 1:20:5 in 20 mM glycine (pH 10.5), 200 mM NaCl, 5% glycerol, and 0.5 mM TCEP, and incubated at room temperature for 6 h.

Single crystals were flash frozen in liquid nitrogen by equilibrating in a cryoprotectant buffer containing the crystallization solution and 25% (v/v) ethylene glycol. X-ray diffraction data were collected at the SER-CAT beamline 22ID of Advanced Photon Source (APS) at Argonne National Laboratory. Crystallographic datasets were first processed with HKL2000^32^. Molecular replacement was performed with PHENIX PHASER module^33^ by using the structure of human SETD3 in complex with SAH and actin H73 peptide (PDB ID 6MBJ) as the search model. Structure refinement was performed with Phenix Refine^34^ with 5% randomly chosen reflections for the validation by Rfree value. COOT^35^ was used for the manual building of structure model and corrections between refinement rounds. Structure quality was analyzed during PHENIX refinements and validated by the PDB validation server. Molecular graphics were generated by using PyMol (Schrödinger, LLC).

### Optimal pH analysis for actin H73 and K73 peptides

To access broad range of pH values, a two-buffer system of varying combination of 10 mM citric acid and 10 mM bis–tris propane (CBTP) was used for pH range below 9, and 20 mM glycine/NaOH was used for high pH above 9.0. For actin H73 (residues 66–80) peptide (Fig. 1b), a reaction mixture containing 20 mM CBTP with varying pH, 50 mM NaCl, 0.1 mg/ml BSA, 1 mM DTT, 0.18 µM SETD3, 20 µM peptide, and 40 µM SAM. The reaction was performed at RT for 1 h. For K73 peptide (Fig. 1d), a reaction mixture containing 20 mM CBTP or 20 mM Glycine/NaOH with varying pH, 50 mM NaCl, 0.1 mg/ml BSA, 1 mM DTT, 15 µM SETD3, 100 µM peptide, and 40 µM SAM. The reaction was performed at 37 °C for 6 h. For both peptides, the reaction was terminated by addition of TFA to a final concentration of 0.4% (v/v), and then diluted 4× with 20 mM Tris (pH 8.0), 50 mM NaCl, 0.1 mg/ml BSA, 1 mM DTT. The methylation activity was measured using the Promega bioluminescence assay (MTase-Glo^TM^)^36^ in which the reaction by-product SAH is converted into ATP in a two-step reaction and ATP can be detected through a luciferase reaction. In general, 5 μL of reaction mixture was transferred to a low-volume 384-well plate and the luminescence assay was performed according to the manufacturer’s protocol. A Synergy 4 Multi-Mode Microplate Reader (BioTek) was used to measure luminescence signal.

### Steady-state kinetic measurement

A reaction mixture contained 20 mM Tris/HCl (pH 8.0) or 20 mM glycine/NaOH (pH 10.5), 50 mM NaCl, 0.1 mg/ml BSA, 1 mM DTT, 0.18–15 µM SETD3 (as indicated in Fig. 3), 40 µM SAM and varying concentration of peptides. The reactions were carried out at room temperature (~22 °C) or 37 °C for different time (20 min to 3 h) with a total volume of 20 µl, and terminated by the addition of TFA to 0.1% (v/v) for Tris/HCl (pH 8.0) or 0.4% (v/v) for Glycine/NaOH (pH 10.5). Samples terminated with the addition of 0.4% (v/v) TFA were then further diluted 4× with buffer 20 mM Tris/HCl (pH 8.0), 50 mM NaCl, 0.1 mg/ml BSA, 1 mM DTT to reduce the TFA concentration. The dependence of the velocity of product formation per enzyme on substrate concentration was analyzed according to the Michaelis–Menten equation.

### Inhibition of sinefungin

For Ki measurement of sinefungin (Supplementary Fig. 2a, b), a reaction mixture contained 20 mM Tris/HCl (pH 8.0), 50 mM NaCl, 0.1 mg/ml BSA, 1 mM DTT, 0.18 µM SETD3, 40 µM H73 (residues 66–80) peptide and first incubated with varied concentration of sinefungin (0–100 µM) for 30 min, reactions were then started by addition of 2–8 µM SAM. After 30 min at room temperature, the reactions were terminated by the addition of TFA to 0.1% (v/v). For comparison of SETD3 and G9a-like protein (GLP) (Supplementary Fig. 2c), a reaction mixture contained 20 mM Tris/HCl (pH 8.0), 50 mM NaCl, 0.1 mg/ml BSA, 1 mM DTT, 0.18 µM SETD3, 40 µM H73 (residues 66–80) peptide, or 2 nM GLP (purified in-house), and 20 µM histone H3 (residues 1–21) peptide. Reaction mixture was first incubated with sinefungin (0–100 µM) for 30 min, and then started by addition of 2 µM SAM. After 10 min at room temperature, the reactions were terminated by the addition of TFA to 0.1% (v/v).

### Activity of SETD3 on H73 and K73 peptides at two temperatures

For H73 (residues 66–80), a reaction mixture contained 20 mM Tris/HCl (pH 8.0), 50 mM NaCl, 0.1 mg/ml BSA, 1 mM DTT, 0.18 µM SETD3, 40 µM SAM, and 20 µM H73 peptide, the reaction was performed for 20 min (Supplementary Fig. 3b). For K73 (residues 66–80), a reaction mixture contained 20 mM Tris/HCl (pH 8.0), 50 mM NaCl, 0.1 mg/ml BSA, 1 mM DTT, 15 µM SETD3, 100 µM SAM, and 700 µM K73 peptide, the reaction was performed overnight (Supplementary Fig. 3c). All reactions were terminated by the addition of TFA to 0.1% (v/v).

### Peptide methylation analysis by mass spectrometry

Actin K73 peptide (residues 66–88) was used as a substrate for SETD3 (Fig. 3d, e). The reaction mixture containing 20 mM glycine/NaOH (pH 10.5), 50 mM NaCl, 0.1 mg/ml BSA, 1 mM DTT, 3 µM SETD3, 10 µM peptide and 40 µM SAM. The reaction was started at 37 °C with a total volume of 80 µl, and was terminated for indicated time in Fig. 3d, e with the addition of 0.4% (v/v) TFA to 10 µl reaction aliquot. Reaction samples were first diluted 5× with a 70:30 water:acetonitrile solution (70% (v/v) acetonitrile) with 0.1% (v/v) TFA. An aliquot of the diluted sample (1 μl) was then mixed with 1 μl of Alpha-CHC (Agilent Technologies) from which 0.6 μl of the mixture was spotted onto the stainless steel MALDI plate. The data was collected using an Applied Biosystems 4700 MALDI-TOF at the MD Anderson Proteomics core facility. A total of 5000 shots were collected per sample using a laser intensity setting of 6000. We note that samples diluted into a 0.1% (v/v) TFA solution in water could not be detected by MALDI-TOF, thus the acetonitrile additive is necessary.

### In vitro methylation reactions on full length actin

GST-β-actin fusion protein was expressed similar to GST-SETD3 (Fig. 3f). Bacteria were lysed by sonication, and cleared lysates were incubated overnight with glutathione sepharose (GE Healthcare) in buffer containing 50 mM Tris pH 7.5, 150 mM NaCl, 0.05% NP-40, 1 mM PMSF. Protein-bound beads were washed with the lysis buffer, and protein was eluted with 100 mM Tris pH 8.0, 10 mg/ml reduced glutathione (Sigma-Aldrich). Purified GST-SETD3 fusion proteins (2 μM) were incubated with GST-β-actin (1 μM) and 2 μCi ^3^H-SAM (American Radiolabeled Chemicals) in a buffer containing 50 mM Tris (pH 8.0), 20 mM KCl, 5 mM MgCl_2_, and 10% glycerol (v/v). Reactions were allowed to proceed overnight at 30 °C. Reactions were resolved by SDS–PAGE, ^3^H-methylation was visualized by autoradiography, and gels were stained with Coomassie blue as a loading control.

### Reporting summary

Further information on research design is available in the Nature Research Reporting Summary linked to this article.

## Supplementary information

Supplementary Information

Reporting Summary

Source Data

## Data Availability

The X-ray structures (coordinates and structure factor files) of SETD3 wild-type and mutant enzymes with bound actin peptide (H73 or K73) have been submitted to the PDB under accession numbers 6OX0 (sinefungin), 6OX2 (methyl-H73), 6OX1 (mixture of H73 and methyl-H73), 6OX3 (K73), 6OX4 (N255A+H73), 6OX5 (N255A+K73). The source data underlying Figs 1b, d, 3a–c and Supplementary Figs. 2 and 3 are provided as a Source Data file. All other data are available from the corresponding author on reasonable request.
