# Supplementary material for: Structural basis for the target specificity of actin histidine methyltransferase SETD3
Source: Nat Commun. 2019 Aug 6;10:3541. doi: 10.1038/s41467-019-11554-6 (PMC6684798; doi:10.1038/s41467-019-11554-6)
Supplement: Supplementary file 1 — Supplementary Information [file 41467_2019_11554_MOESM1_ESM.pdf]

Supplementary Information

Dai et al.

Structural basis for the target specificity of actin histidine methyltransferase SETD3

Shaobo Dai[1], John R. Horton[1], Clayton B. Woodcock[1], Alex W. Wilkinson[2], Xing Zhang[1], Or Gozani[2], Xiaodong Cheng[1,*]

[1]Department of Epigenetics and Molecular Carcinogenesis, The University of Texas MD Anderson Cancer Center, Houston, Texas, USA

[2]Department of Biology, Stanford University, Stanford, CA, USA

*e-mail: xcheng5@mdanderson.org

All e-mail addresses:

SD (SDai@mdanderson.org)

JRH (JRHorton@mdanderson.org)

CBW (CBWoodcock@mdanderson.org)

AWW (alexw2@stanford.edu)

XZ (XZhang21@mdanderson.org)

OG (ogozani@stanford.edu)

XC (xcheng5@mdanderson.org)

Three figures and 1 Table

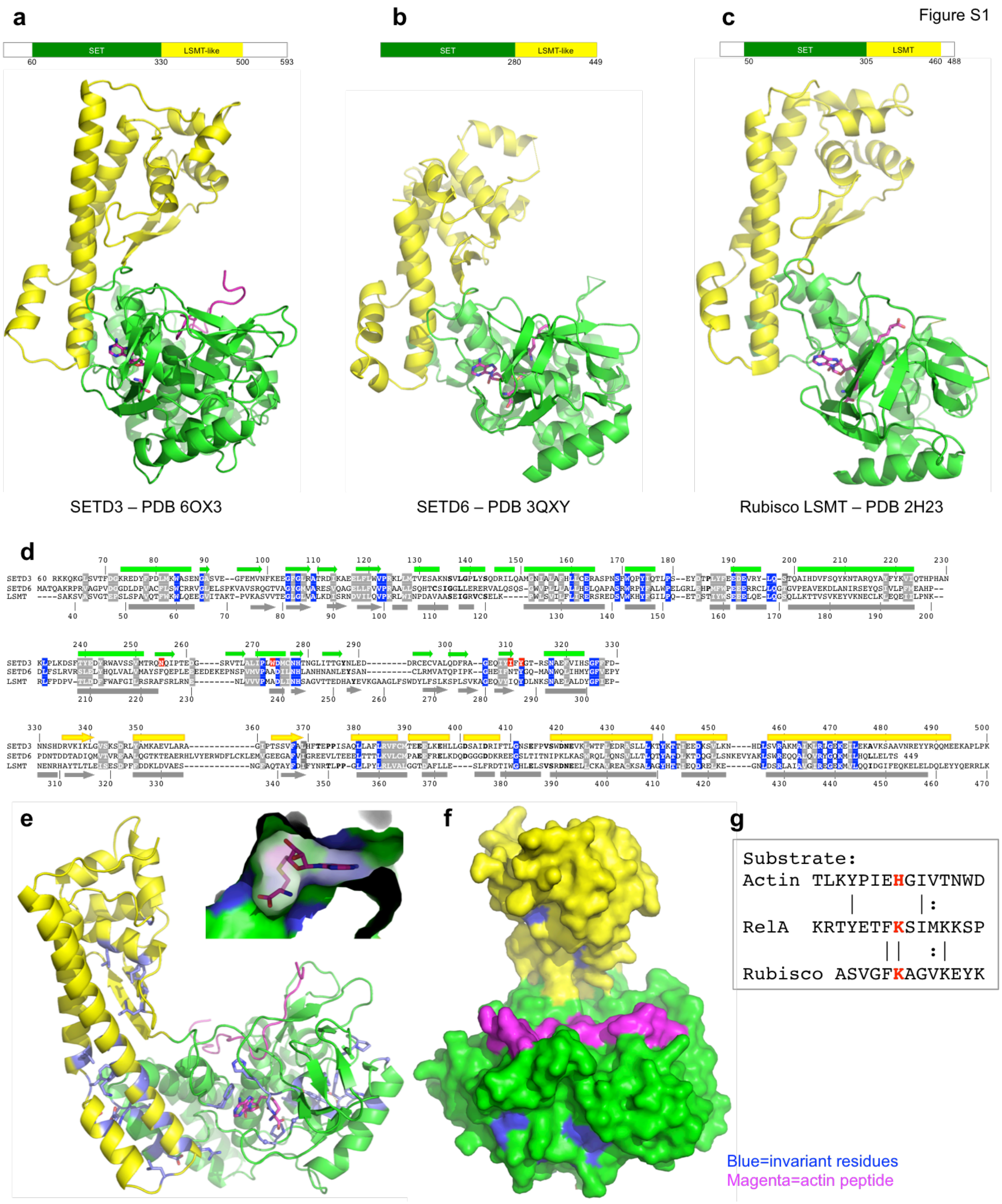

Supplementary Fig. 1 | SETD3, SETD6, and LSMT have conserved structures. **a-c**, Structures of three enzymes with SET domains in green and helical domains in yellow. **d**, Sequence alignment of there enzymes with invariant residues (white letters in blue background)

and conserved residues (white letters in grey background) [SETD3: UniProtKB/Swiss-Prot sequence Q86TU7.1; SETD6: NCBI reference sequence NP_079136.2; LSMT: UniProtKB/Swiss-Prot sequence Q43088.1]. The four amino acids in red background are the active-site residues in SETD3. **e**, SETD3 structure showing the invariant residues involved in structural integrity, mostly intra-molecular interactions in the interior of the molecule conferring stability. Invariant residues form a pocket of binding SAM (shown in stick model). **f**, Surface representation showing surface of substrate binding (actin peptide in magenta) has few invariant residues (blue). **g**, Three protein substrates with limited sequence similarity immediately surrounding the methylation target (in red).

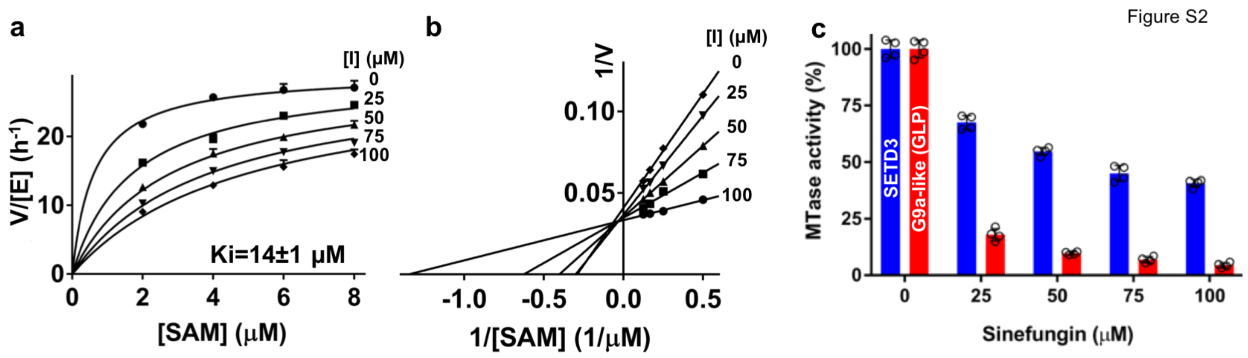

Supplementary Fig. 2 | Sinefungin as a SAM-competitive inhibitor. **a-b**, Ki measurement of sinefungin against SAM and corresponding Lineweaver-Burk plot for SETD3 (panel b). **c**, Relative methyltransferase activities of SETD3 (on active H73 peptide) and GLP (on histone H3 K9 peptide) in the presence of sinefungin. Data represent the mean ± SD of N number of independent determinations (N=2 in panels a and b and N=4 in panel c) performed in duplicate. Source data are provided as a Source Data file.

Figure S3

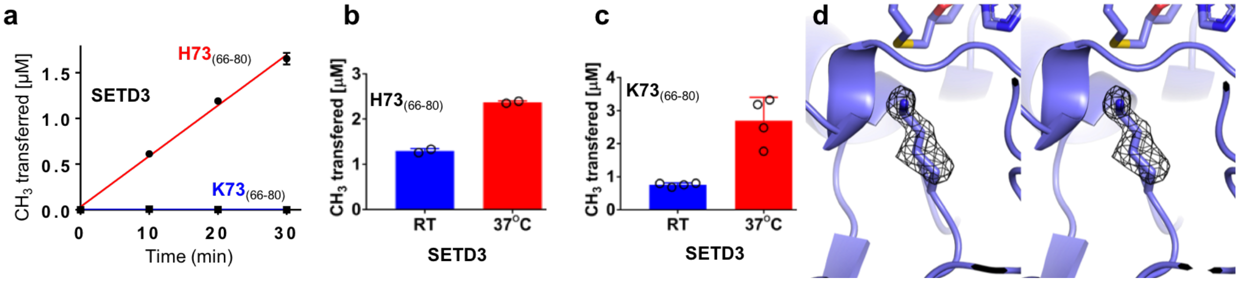

Supplementary Fig. 3 | **a**, SETD3 is inactive on $K73_{(66\text{-}80)}$ peptide (blue peptide) under the conditions optimized for $H73_{(66\text{-}80)}$ (red line): [E]=0.18 µM, [SAM]=40 µM, [S]=20 µM, pH 8.0 at room temperature for indicated reaction time. **b**, SETD3 activity on $H73_{(66\text{-}80)}$ at two difference temperatures under the conditions of [E]=0.18 µM, [SAM]=40 µM, [S_{H73}]=20 µM at pH 8.0 for 20 min reaction. **c**, SETD3 activity on $K73_{(66\text{-}80)}$ at room temperature (~22 °C) and 37 °C under the conditions of increased amounts of [E]=15 µM, [SAM]=100 µM and [S_{K73}]=700 µM at pH 8.0 for overnight reaction. Data represent the mean ± SD of N number of independent determinations (N=2 in panels a and b and N=4 in panel c) performed in duplicate. Source data are provided as a Source Data file. **d**, A stereo image of omit electron density (Fo-Fc) omitting the side chain of K73 in the active site contoured at 4σ above the mean.

Supplementary Table 1. Summary of X-ray data collection at SERCAT 22-ID beamline (wavelength=1Å) and refinement statistics (*)

PDB ID	6OX0	6OX2	6OX1	6OX3	6OX4	6OX5
SETD3	WT	WT	WT	WT	N255A	N255A
Actin peptide	$H73_{(66-80)}$	$meH73_{(66-80)}$	$H73/meH73_{(66-80)}$	$K73_{(66-88)}$	$H73_{(66-80)}$	$K73_{(66-88)}$
Cofactor	Sinefungin	SAH	SAH	SAH	SAH	SAH
Space group	$P2_1$	$P2_1$	$P2_1$	$P2_1$	$P2_1$	$C222_1$
Cell dimensions (Å)	60.27, 175.19, 66.01	60.10, 175.94, 66.26	60.31, 175.87, 66.97	60.23, 175.75, 66.47	60.39, 177.04, 65.91	60.47, 116.50, 174.66
$\alpha=\gamma=90°$, β (°)	92.8	92.5	92.4	92.4	92.5	90
Resolution (Å)	41.68-1.75 (1.81-1.75)	43.57-2.09 (2.16-2.09)	31.27-1.95 (2.02-1.95)	36.65-1.79 (1.85-1.79)	36.73-2.29 (2.37-2.29)	34.94-2.09 (2.16-2.09)
[a] R_{merge}	0.135 (0.965)	0.142 (0.911)	0.146 (0.822)	0.253 (0.983)	0.247 (0.682)	0.158 (1.022)
R_{pim}	0.033 (0.444)	0.060 (0.443)	0.083 (0.499)	0.089 (0.654)	0.112 (0.437)	0.036 (0.450)
$CC_{1/2}$, CC	(0.627, 0.878)	(0.665, 0.894)	(0.461, 0.794)	(0.419, 0.768)	(0.409, 0.762)	(0.384, 0.745)
[b] $\langle I/\sigma I \rangle$	19.7 (1.9)	12.6 (1.6)	11.7 (2.9)	9.2 (1.7)	8.8 (1.6)	18.5 (2.2)
Completeness (%)	98.0 (89.8)	97.0 (91.5)	98.3 (99.4)	98.4 (93.6)	95.2 (75.0)	96.6 (82.9)
Redundancy	15.9 (12.6)	6.1 (4.0)	3.7 (3.3)	7.1 (2.5)	5.0 (2.8)	16.1 (5.5)
Observed reflections	2,107,016	476,569	363,024	910,609	293,503	564,798
Unique reflections	132,568 (12,128)	78,578 (7,372)	99,091 (9,993)	127,675 (12,113)	58,449	35,098
**Refinement**	2 complexes	2 complexes	2 complexes	2 complexes	2 complexes	1 complex
Resolution (Å)	1.75	2.09	1.95	1.79	2.29	2.09
No. reflections	132,006	78,478	98,907	127,513	58,365	35,025
[c] R_{work} / [d] R_{free}	0.205 / 0.225	0.199 / 0.240	0.171 / 0.200	0.208 / 0.247	0.193 / 0.232	0.168 / 0.205
No. Atoms						
Protein	7803	7856	7881	7826	7775	3857
Peptide	240	246	270	302	237	144
Cofactor	54	52	52	52	52	26
Solvent	761	612	842	774	605	267
B Factors ($Å^2$)						
Protein	36.3	45.8	29.8	28.5	42.4	39.7
Peptide	44.9	78.1	37.0	36.9	54.4	50.6
Cofactor	20.4	27.2	16.2	15.9	26.5	21.4
Solvent	43.6	49.8	38.6	36.9	49.0	44.9
**R.m.s. deviations**						
Bond lengths (Å)	0.002	0.002	0.006	0.004	0.002	0.002
Bond angles (˚)	0.5	0.4	0.8	0.7	0.5	0.5

* Values in parenthesis correspond to highest resolution shell.

[a] $R_{merge}=\Sigma \mid I - \langle I \rangle \mid / \Sigma I$, where I is the observed intensity and $\langle I \rangle$ is the averaged intensity from multiple observations.

[b] $\langle I/\sigma I \rangle$ = averaged ratio of the intensity (I) to the error of the intensity (σI).

[c] $R_{work}=\Sigma \mid Fobs - Fcal \mid / \Sigma \mid Fobs \mid$, where Fobs and Fcal are the observed and calculated structure factors, respectively.

[d] R_{free} was calculated using a randomly chosen subset (5%) of the reflections not used in refinement.
