# Supplementary material for: Structural basis for the target specificity of actin histidine methyltransferase SETD3
Source: Nat Commun. 2019 Aug 6;10:3541. doi: 10.1038/s41467-019-11554-6 (PMC6684798; doi:10.1038/s41467-019-11554-6)
Supplement: Supplementary file 2 — Reporting Summary [file 41467_2019_11554_MOESM2_ESM.pdf]

nature research

Corresponding author(s): Xiaodong Cheng

Last updated by author(s): Jul 19, 2019

Reporting Summary

Nature Research wishes to improve the reproducibility of the work that we publish. This form provides structure for consistency and transparency in reporting. For further information on Nature Research policies, see Authors & Referees and the Editorial Policy Checklist.

Statistics

For all statistical analyses, confirm that the following items are present in the figure legend, table legend, main text, or Methods section.

n/a	Confirmed	
☐	☒	The exact sample size (*n*) for each experimental group/condition, given as a discrete number and unit of measurement
☐	☒	A statement on whether measurements were taken from distinct samples or whether the same sample was measured repeatedly
☒	☐	The statistical test(s) used AND whether they are one- or two-sided<br>*Only common tests should be described solely by name; describe more complex techniques in the Methods section.*
☒	☐	A description of all covariates tested
☒	☐	A description of any assumptions or corrections, such as tests of normality and adjustment for multiple comparisons
☐	☒	A full description of the statistical parameters including central tendency (e.g. means) or other basic estimates (e.g. regression coefficient) AND variation (e.g. standard deviation) or associated estimates of uncertainty (e.g. confidence intervals)
☒	☐	For null hypothesis testing, the test statistic (e.g. *F*, *t*, *r*) with confidence intervals, effect sizes, degrees of freedom and *P* value noted<br>*Give P values as exact values whenever suitable.*
☒	☐	For Bayesian analysis, information on the choice of priors and Markov chain Monte Carlo settings
☒	☐	For hierarchical and complex designs, identification of the appropriate level for tests and full reporting of outcomes
☒	☐	Estimates of effect sizes (e.g. Cohen's *d*, Pearson's *r*), indicating how they were calculated

Our web collection on statistics for biologists contains articles on many of the points above.

Software and code

Policy information about availability of computer code

Data collection	X-ray diffraction data were collected from at the SER-CAT beamline 22ID of Advanced Photon Source (APS) at Argonne National Laboratory.
Data analysis	Crystallographic datasets were first processed with HKL2000.<br>Molecular replacement was performed with PHENIX PHASER module.<br>Structure refinement was performed with Phenix Refine with 5% randomly chosen reflections for the validation by Rfree value.<br>COOT was used for the manual building of structure model and corrections between refinement rounds.<br>Structure quality was analyzed during PHENIX refinements and validated by the PDB validation server.<br>Molecular graphics were generated by using PyMol (Schrödinger, LLC).

For manuscripts utilizing custom algorithms or software that are central to the research but not yet described in published literature, software must be made available to editors/reviewers. We strongly encourage code deposition in a community repository (e.g. GitHub). See the Nature Research guidelines for submitting code & software for further information.

Data

Policy information about availability of data

All manuscripts must include a data availability statement. This statement should provide the following information, where applicable:

- Accession codes, unique identifiers, or web links for publicly available datasets
- A list of figures that have associated raw data
- A description of any restrictions on data availability

The X-ray structures (coordinates and structure factor files) of SETD3 wild-type and mutant enzymes with bound actin peptide (H73 or K73) have been submitted to the PDB under accession numbers 6OX0 (sinefungin), 6OX2 (methyl-H73), 6OX1 (mixture of H73 and methyl-H73), 6OX3 (K73), 6OX4 (N255A+H73), 6OX5 (N255A +K73).

October 2018

Field-specific reporting

Please select the one below that is the best fit for your research. If you are not sure, read the appropriate sections before making your selection.

☒ Life sciences ☐ Behavioural & social sciences ☐ Ecological, evolutionary & environmental sciences

For a reference copy of the document with all sections, see nature.com/documents/nr-reporting-summary-flat.pdf

Life sciences study design

All studies must disclose on these points even when the disclosure is negative.

Sample size	As described in Figures 1 (pH ranging from 5 to 11) and Figure 3 (variation of peptide concentrations)
Data exclusions	None
Replication	Kinetic data represents the mean +/- SD of N number of independent determinations performed for the wild type enzyme (N=3 in Fig. 3a), mutant enzymes (N=2), activities on lysine substrate (N=4 in Supplementary Fig. 3c) and inhibition (N=4 in Supplementary Fig. 2c).
Randomization	No randomization was necessary as only single variable changed per experiment
Blinding	Blinding was not necessary as measurements were performed using discrete and quantitative assays

Reporting for specific materials, systems and methods

We require information from authors about some types of materials, experimental systems and methods used in many studies. Here, indicate whether each material, system or method listed is relevant to your study. If you are not sure if a list item applies to your research, read the appropriate section before selecting a response.

Materials & experimental systems

n/a	Involved in the study	
☒	☐	Antibodies
☒	☐	Eukaryotic cell lines
☒	☐	Palaeontology
☒	☐	Animals and other organisms
☒	☐	Human research participants
☒	☐	Clinical data

Methods

n/a	Involved in the study	
☒	☐	ChIP-seq
☒	☐	Flow cytometry
☒	☐	MRI-based neuroimaging
